# A shift from anaerobic digestion to dark fermentation in glycol ethylene fermentation

**DOI:** 10.1007/s11356-020-12149-1

**Published:** 2021-02-09

**Authors:** Gaweł Sołowski, Tadeusz Ziminski, Adam Cenian

**Affiliations:** Institute of Fluid-Flow Machinery of Polish Academy of Sciences, Gdańsk, Poland

**Keywords:** Bacteria rests, Petrochemical wastes, Hydrogen, Methane, Hydrogen sulphide, Unpretreated inoculum

## Abstract

Anaerobic digestion of aqueous glycol ethylene was tested. The process lasted two cycles of 7 days, but after the second cycle, high hydrogen production occurred shift to dark fermentation. The biogas production lasted 14 days, obtaining peak values of hydrogen, and then rapidly stopped. In investigations, the following were checked: dependence of hydrogen, methane and hydrogen sulphide in the process. Mixtures of water with glycol ethylene mass ratio from 0.6 to 0.85 were substrates in experiments. The highest methane production was for water ethylene 0.7 ratio 2.85 L of methane with a yield of 178 mL of methane/g VSS (volatile suspended solids) of glycol ethylene. The optimal ratio of water and glycol ethylene was 0.85 25.5 mL of hydrogen (giving yield 1.71 mL of hydrogen/g VSS of glycol ethylene) and 1.71 mL of hydrogen sulphide emission for a 0.6 ratio. Popular polymer industry wastes, glycol ethylene, can be utilised by anaerobic digestion.

## Introduction

Glycol ethylene is a relevant part of waste from fuel production and plastics (Walker and Rothman 2020). The utilisation of this waste is sufficient by being toxic to aqueous life (Zheng et al. 2017; Villa Montoya et al. 2019). Bacterial methods of removal of this pollution are quite promising (Millati et al. 2020). Anaerobic digestion (AD) is transforming organic matter by bacteria into methane (Fagbohungbe et al. 2019). There were also ideas to use as enrichment for some ceramics, but they demanded high amounts of energy (Kaur et al. 2019). Anaerobic digestion requires significantly lower temperatures between 30 and 60 °C (depending on bacteria type psychro, meso, or thermophilic (Chasnyk et al. 2015)). The satisfying results had composting of wastewater with ethylene glycol also but without a report of valuable products (Qi et al. 2020). There are also ideas of use ethylene glycol as a substrate for hydrogen production by reforming, but the method requires enormous costs of energy in comparison to AD. One of the approaches was the conversion of ethylene glycol to methane (Elreedy et al. 2017) by anaerobic digestion. The studies investigated AD as part of petrochemical wastewater (Elreedy et al. 2015) or polyester wastes (Shin and Bae 2019). There were no published data of anaerobic digestion on glycol ethylene only solution. Elreedy et al. (2019) reported hydrogen production and methane from ethylene glycol wastewater in psychrophilic conditions but without comparing it with mesophilic conditions. Other works do not concentrate on methane efficiency but on toxicity measurements and acetic acid synthesis (Stewart et al. 1995). Experiments with only water-ethylene glycol can show the influence of other pollutants for overall ethylene digestion (Tan et al. 2020). Elreedy et al. (2015, 2017) observed hydrogen and methane production from the AD of petrochemical wastewater, but there was no mention if in biogas hydrogen sulphide emission occurred. The occurrence of the emission can determine the origin of hydrogen sulphide without the addition of the source of the compound in feed, and then the gas would be caused by the digestion of bacteria rests. It also is not determined if hydrogen production depends or not on hydrogen sulphide emission. Then it is worth checking the dependencies between these two gases.

## Materials and methods

### Inoculum

The inoculum used for the research came from a mesophilic biogas plant at Lubań (Pomerania), fed mainly manure and maize silage (see Table 1). The bacteria were not extra supplemented by any nutrients; they were only mixed with a substrate in 2-L reactors with a working volume of 1.2 L.

**Table 1:** Comparison of yield in different water:glycol ethylene ratio

Substrate	pH	TS	VSS	H_2_ yield with error ± 0.04% (mL H_2_/g VSS)	CH_4_ yield with error ± 0.04% (L CH_4_/g VSS)	H_2_S yield with error ± 0.04% (mL H_2_S/g VSS)	Reference
Inoculum coming from biogas plant sludge	7.84	1.5% ± 0.03%	37.01% TS ± 1.22%	0	0	0	This study
Water:glycol ethylene ratio of 0.6 with inoculum	7.19	1.54% ± 0.03%	37.21% TS ± 1.23%	1.489	0.128	0.11	This study
Water:glycol ethylene ratio of 0.6	5.66	3.08% ± 0.03%	46.63% TS ± 1.02%	0	0	0	This study
Water:glycol ethylene ratio of 0.7 with inoculum	7.52	1.63% ± 0.03%	37.64% TS ± 1.20%	1.403	0.178	0.007	This study
Water:glycol ethylene ratio of 0.7	5.9	6.8% + 0.02%	48.22% TS ± 1.12%	0	0	0	This study
Water:glycol ethylene ratio of 0.65 with inoculum	7.34	1.59% + 0.02%	37.33% TS ± 1.22%	1.527	0.146	0.0024	This study
Water:glycol ethylene ratio of 0.65	5.74	3.1% ± 0.02%	48.2% TS ± 1.2%	0	0	0	This study
Water:glycol ethylene ratio of 0.85 with inoculum	7.67	1.72% ± 0.02%	38.21% TS ± 1.2%	1.71	0.161	0.002	This study
Water:glycol ethylene ratio of 0.85	6.2	7.1% ± 0.03%	60.1% TS ± 1.05%	0	0	0	This study
Water:glycol ethylene ratio of 0.75 with inoculum	7.6	1.69 + 0.03%	37.91% TS ± 1.20%	0.0988	0.164	0.003	This study
Water:glycol ethylene ratio of 0.75	6.12	5.1% + 0.02%	56.31 %TS ± 1.02%	0	0	0	This study
Water:glycol ethylene ratio of 0.63 with inoculum	7.24	1.56 + 0.02%	37.27% TS ± 1.06%	1.01	0.085	0.103	This study
Water:glycol ethylene ratio of 0.63	5.69	2.5% + 0.04%	47.1%TS ± 1.03%	0	0	0	This study
Synthetic petrochemical waste with glycol ethylene 13.27 g COD/L/day heat-treated inoculum	7.0	39.59 ± 1.02	26.46 ± 1.55	47.55	0.151	-	(Elreedy et al. 2017)
Synthetic petrochemical waste with glycol ethylene 1.67 g COD/L/day anaerobic sequencing reactor heat-treated inoculum	5.0–5.5	32.98 ± 0.93	22.33 ± 0.66	189.9	0.132		(Elreedy et al. 2019)
Coke after Fe autoclave pretreatment 10 g TS/L	7.57	Not available	Not available		0.257		(Yang et al. 2020)
Maize silage 3.7 g VSS/L	7.5	33%	95%		0.63		(Molino et al. 2019)
A mixture of hydrothermally pretreated asbestos to glucose in proportion 1:6 36.16 g VSS (asbestos 5 g VSS/L, glucose 31.16 g VSS/L) Activated sludge with stressing using heat–shock 105 C for 1 h	5.45	Not available	Not available	1.3			(Spasiano 2018)

### Substrates

Mixtures of water with glycol ethylene with mass ratio from 0.6 to 0.85 of solution were added to the reactor to obtain a concentration of a substrate 11 mL/L. Inoculum concentration in the reacting mixture was 0.97 L of inoculum/L. The next feeding of reactors was after 7 days as biogas stopped. The addition of 11 mL aqueous glycol ethylene was in the same ratios as earlier. Anaerobic conditions were obtained by the removal of air using carbon dioxide.

Table 1 presents ratios of water to glycol ethylene and the characteristics of substrates and inoculum. The characteristics were prepared due to standards of NREL (National Renewable Energy Laboratory) of biogas (Moriarty 2013) and biomass characteristics (Hames et al. 2008). By these standards were measured total solids (TS) and volatile suspension solids (VSS) called dry organic mass (Fagbohungbe et al. 2019) also. The pH of the process was measured at the beginning of the experiment. pH was between 7.49 and 7.9 depending on the ratio of water to glycol ethylene. Fermentations were cultivated at 37 °C temperature applied in a biogas plant and glycerol fermentation (Pachapur et al. 2019).

### Biogas content

Biogas increase was measured using the Owen method (Logan et al. 2002). The biogas was passed from outlets of reactors through polypropylene ducts to cylinders with water with few drops of barrier layer at the top. The barrier layer was made from a mixture of diesel oil and popular dish detergent Ludwik® in mass ratio 1:10. The volume of stuffed water is the volume of the produced biogas. Volumes of biogas were calculated by using Eq. (1) where *V*_s_ is the volume of measured gas at standard temperature and pressure, *V*_m_ is the volume of measured gas at ambient conditions, *T*_m_ is the ambient temperature, *T*_s_ is the standard temperature and *P*_s_ is the standard pressure.

1 $$ {V}_{\mathrm{s}}=\frac{V_{\mathrm{m}}\bullet {T}_{\mathrm{s}}\bullet {P}_{\mathrm{m}}}{T_{\mathrm{m}}\bullet {P}_{\mathrm{s}}} $$

**Fig. 1:**
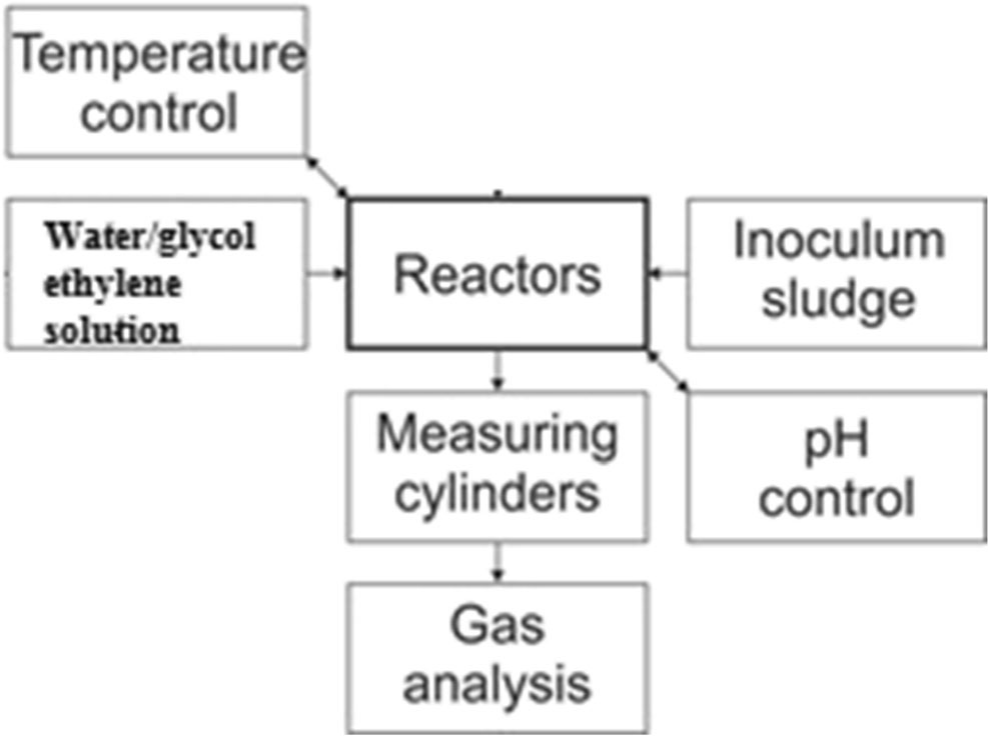
Scheme of the experimental setup

The qualitative and quantitative assessments of the gases were performed like in the earlier article (Sołowski et al. 2020a). A portable biogas analyser (GA5000, Geotech) measured the volume of biogas in the cylinder being at least 0.45 dm^3^. The device posed ATEX II 2G Ex ib IIA *T*_1_ *G*_b_ (*T*_a_ from − 10 to + 50 °C), IECEx and CSA quality certifications, and UKAS ISO 17025 calibration certificate. The equipment measured the following biogas components (ranges): CH_4_ (from 0 to 100%), CO_2_ (from 0 to 100%), O_2_ (from 0 to 25%), H_2_ (from 0 to 1000 ppm) and H_2_S (from 0 to 5000 ppm). The device was calibrated twice a week. If the hydrogen concentration was above 1000 ppm, the gas was measured using a gas chromatograph (GC) with a thermal conductivity detector (TCD) in the second stage. Argon was a carrier (gas flow rate was 0.6 mL/h). A Silco packed column Restek® with characteristics (2 m/2 mm ID 1/8″ OD Silica) was used. Besides reactors with glycol ethylene, there were three reactors with the only inoculum. The scheme of research equipment is shown in Fig. 1.

### Statistical analysis

All experiments were triplicated. Tables and figures contain the mean values for biogas measurements, inoculum and substrate characteristics. Biogas analysis was carried minimum of twice, giving an error of yield of 0.04%.

## Results and discussion

### Methane production

**Fig. 2:**
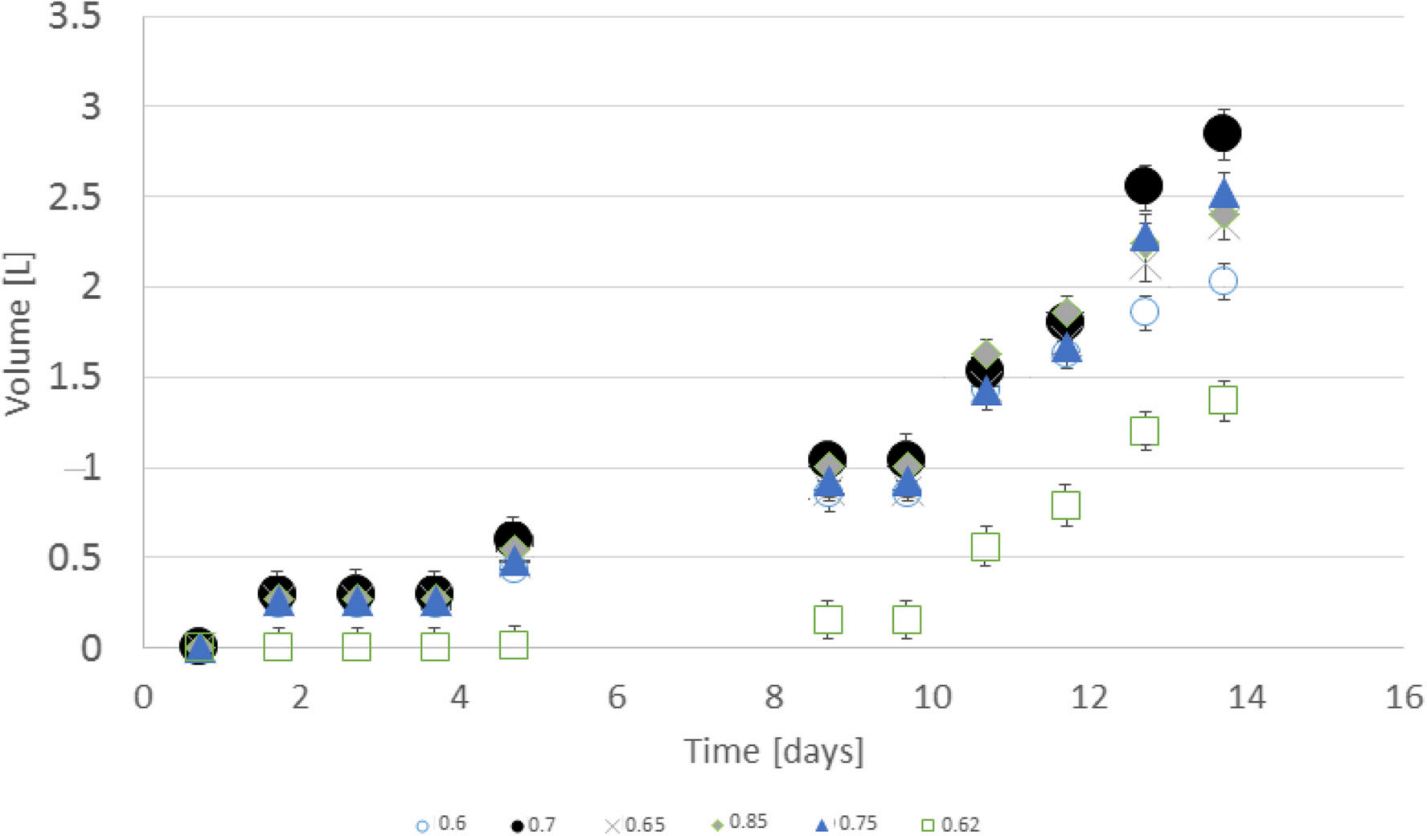
Cumulative methane production from water-glycol ethylene solution from ratio 0.6 to 0.85

Figure 2 shows accumulated methane production results.

The first period of methane production lasted 5 days. Then after the second cycle, it produced from the 9th to 14th days. The biogas production break between feeding and first emission was 1 day. The highest overall methane production was for 0.7 ratios. In the rest of the ratios, methane production varied very rapidly during the days. The length of fermentation was identical in all cases. The methane production in the second cycle doubled in comparison to the first part. The change of initial concentration of glycol ethylene from 0.6 to 0.8 shows that a ratio of 0.7 was optimal from the view of volumetric methane yield. A 0.7 proportion was the most efficient in the range from 0 to the 10th day and from the 13th to 14th day. From the 11th to 12th day, methane production was the highest for 0.85 proportions. On the 13th day, it could be discerned that there is a slower increase of methane production in 0.85 ratios (0.6 L of methane) than 0.75 (growth was 1 L of methane in 1 day). Methane production was decreasing rapidly above proportions 0.75 in comparison to abating ratios from 0.65 to 0.7. Nevertheless, in the case of ratios of 0.85, the methane yield increased again. At 0.62 was a rapid decrease in methane production, while at 0.65, it increased. At 0.85, methanogenesis was slightly better than at the level of 0.65. Such differences did not occur in fats (Nguyen et al. 2019) or wheat straw (Byrne et al. 2018). The reactors with inoculum only did not produce any biogas.

### Hydrogen production

**Fig. 3:**
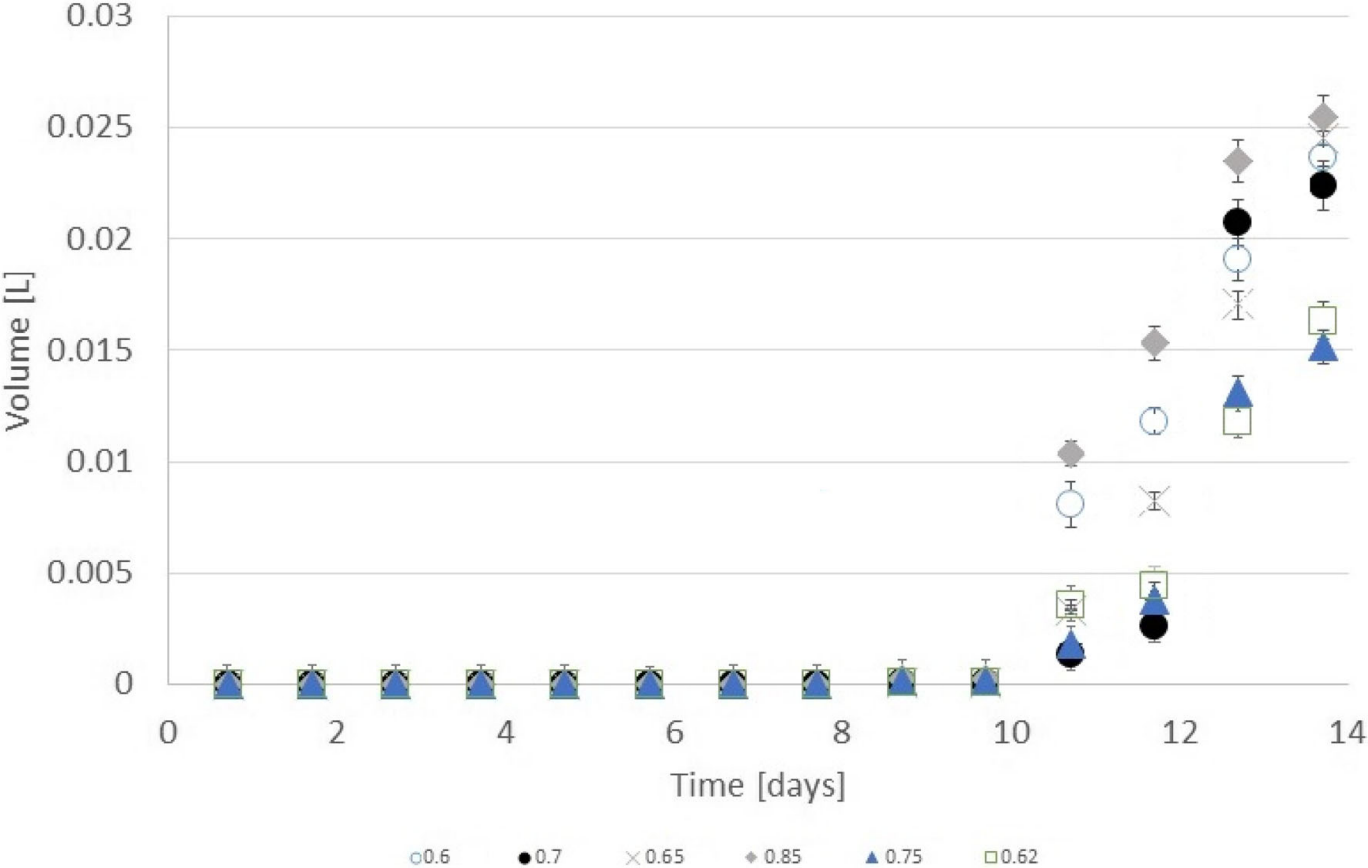
Cumulative hydrogen production from water-glycol ethylene solution from ratio 0.6 to 0.85

Hydrogen production occurred in significant volumes after the second cycle on the 11th day (4 days after addition). In this period, hydrogen production was high enough for the classification of the process as dark fermentation (Detman et al. 2018). Relevant simultaneous methane presence caused what some researchers call such dark fermentation hydrogenotrophic anaerobic digestion (Mirmohamadsadeghi et al. 2021). The differences in overall cumulative hydrogen production and yields (see in Fig. 3 and Table 1) were related to the initial concentration of glycol ethylene. In every ratio, hydrogen production stopped after a sudden increase in daily production at the end of the 14th day of the experiment. Cumulative hydrogen production was optimal in the exterior ratios of 0.6 and 0.85 water to glycol ethylene. In the last 2 days of the research occurred a remarkable change. A significant change occurred in the final 2 days of the experiment. The hydrogen production trends changed every day in the utmost 3 days in many ratios. In ratios 0.7, hydrogen production was the least from all until the 12th day. In proportion 0.7, the hydrogen production growth changed after every day of emission. An interesting point was at the end of the 13th day. On that day, hydrogen production from 0.85 was slightly worse than 0.7. At the same time, there was a rapid burst of hydrogen higher than in other proportions of water-glycol ethylene. On this day, the hydrogen volume increased by 20 mL (0.6 ratios) while the next ratios 5 mL (0.65). The 0.7 proportion was the 4th highest overall hydrogen production also on the 13th day. On the 14th day, the increase of hydrogen production for 0.65 water-glycol ethylene was 5 mL, while for ratios 0.65–7 mL of hydrogen. At the same point for 0.7 ratios, it was just 2 mL of hydrogen growth. The overall cumulative hydrogen production after 14 days and the highest yield was for 0.85 ratios. Though, the highest cumulative hydrogen volume was for 0.6 water-glycol ethylene. The least hydrogen production was usually ratios 0.75 beside the 12th day that was for proportion 0.7. Trends of growth of hydrogen production from glycol ethylene were specifically not observed in other substrates like glucose (Argun and Onaran 2018), cotton (Sołowski et al. 2020a), sour cabbage (Sołowski et al. 2019) or wheat straw (Ghimire et al. 2018).

### Hydrogen sulphide emission

**Fig. 4:**
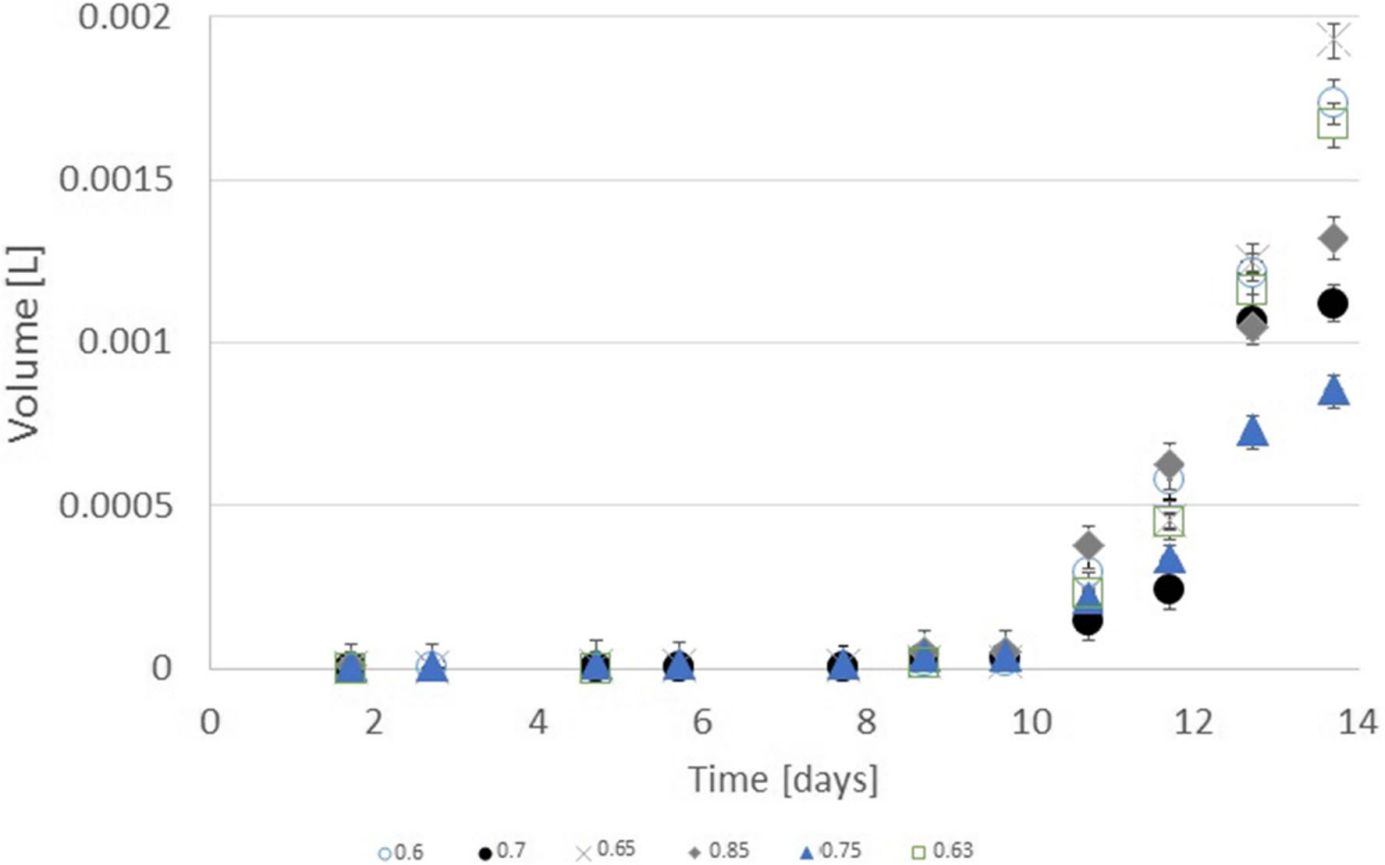
Cumulative hydrogen sulphide emission from water-glycol ethylene solution from ratio 0.6 to 0.85

**Fig. 5:**
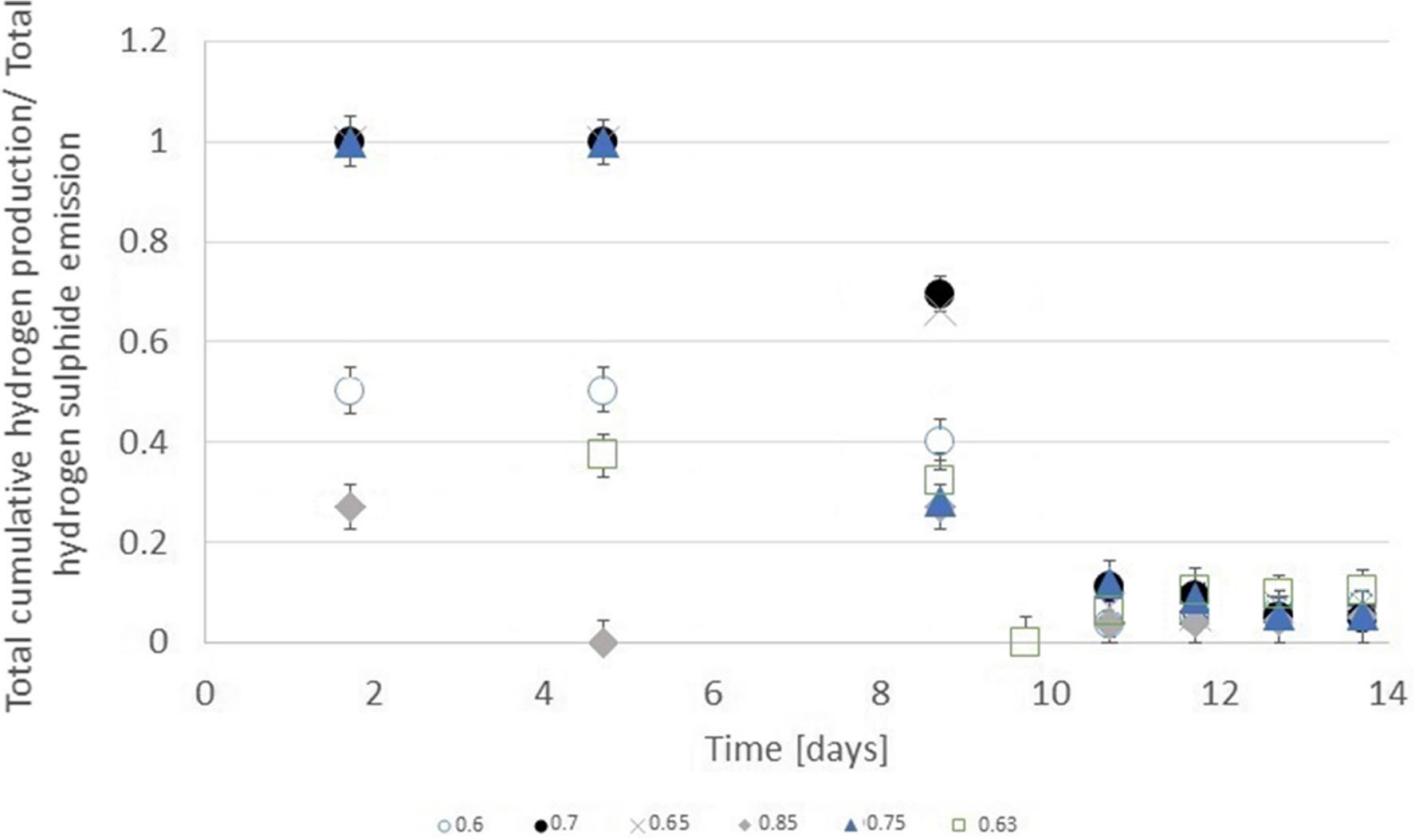
Total cumulative hydrogen production/total hydrogen sulphide emission from water-glycol ethylene solution from ratio 0.6 to 0.85

The hydrogen sulphide emission had similar trends of growth to hydrogen production but several times less. Sołowski et al. (2020b) observed the presence of coinciding with these two gases. Lee et al. (2019) observed common hydrogen sulphide emission in anaerobic digestion. Shivasankaran et al. (2020) discerned that this emission occurs mostly in high protein substrate due to the necessity of breaking amino acids with sulphur during fermentation. Thus, it originated from bacteria rests. Values of ratio (water-ethylene glycol) with the highest hydrogen sulphide emission were different from hydrogen and methane. The hydrogen sulphide originates from protein (from bacteria rests). In the feed, there were not any added compounds with sulphur, so the only source of hydrogen sulphide was from the decomposition of inoculum parts. The bacteria, during the biogas production, digested substrate with bacterial rest that contained proteins. No biogas at prime samples proved that glycol ethylene increased the digestion of the proteins from bacteria rests. Therefore, methionine, cysteine, or both amino acids with sulphur present in bacteria proteins were decomposed with the emission of hydrogen sulphide. The hydrogen sulphide emission was the highest for the ratio of 0.65 (see Fig. 4). Promnuan et al. (2020) reported biohythane–hydrogen production and then methane but without the existence of both gases at the same time. Hydrogen sulphide emission was not discernible up to the 11th day. Figure 5 shows that the differences between total hydrogen production and total hydrogen sulphide emission were not stable until this day. In the first period made after the 11th day, the ratio between the two gases was stable. Therefore, besides the 9th day, a ratio of the hydrogen sulphide emission to hydrogen production was proportional. The value of the proportion depended on the dilution of the substrate. These critical points of difference were the result of the digestion of new portion of aqueous glycol ethylene. After 10 days, it can be discerned that the increase of hydrogen production in comparison to hydrogen sulphide was stable. Thus, hydrogen production was coherent with hydrogen sulphide emission. It used bacteria rests, acting as an activator for hydrogen production with hydrogen sulphide emission being the result of the decomposition of proteins during this digestion.

### Overall discussion

Table 1 shows the yields of three investigated gas. The already published approaches with petrochemical wastes showed 200 times higher hydrogen production but 30 less methane production (Elreedy et al. 2019). The study is not a popular research topic. There was no published research investigating the dependence of hydrogen production on hydrogen sulphide emission. Another relevant case showed that for efficient methane production, it was not necessary to supplement nutrients like in Aworanti et al.’s work (Aworanti et al. 2017). The high hydrogen production did not occur as in Elreedy et al. (2017) because the research was only glycol ethylene without petrochemical wastes. In this study, glycol ethylene worked as a substrate and as chemical pretreatment also (Hu and Chen 2007; Gyanashree and Jyotirekha 2018). Aqueous glycol ethylene addition transformed the methane process into hydrogen production. The glycol ethylene with water shifted slowly from methane production to slight hydrogen occurrence in the last days of the process. Hydrogen production was unusually similar to sour cabbage (Sołowski et al. 2019), the highest in the final 2 days of the process, not as usual in the first hours of fermentation (Cazier 2015; Motte et al. 2015). Proportions between accumulated hydrogen production and hydrogen sulphide emission were often stable. These trends were not observed in previous publications of dark fermentation (Mechery et al. 2019) or anaerobic digestion (Gallipoli et al. 2020). This state is caused by analysing most samples from AD and DF only in GC. Simultaneous determination of hydrogen and hydrogen sulphide needs to combine different techniques of GC analysis. Both gases could be detected periodically in GC, but their dependencies had not been supposed. Thus, it was not keen to be tested because it seemed to be aimless.

## Conclusions

Hydrogen sulphide did not come from substrates, but as a metabolite of digestion of bacteria rests. Hydrogen sulphide emission and hydrogen production were proportional. The proportions were broken during feeding for 2 days until the reacting mixture stabilised. The highest methane production was for water ethylene 0.7 ratio 2.85 L of methane (yield 178 mL of methane/g VSS of glycol ethylene). The apical hydrogen production was for ratio 0.85 25.5 mL of hydrogen (yield 1.71 mL of hydrogen/g VSS of glycol ethylene), and hydrogen sulphide emission for 0.6 ratios 1.71 mL (yield 0.11 mL of hydrogen sulphide/g VSS of glycol ethylene). Aqueous glycol ethylene is a good source for methane production. Anaerobic digestion can be used as pretreatment in petrochemical wastes, but after reaching some glycol ethylene concentration, the process would be turning into dark fermentation.

## Data Availability

All data of the study can be shared after sending a request to the corresponding author.
